# Exogenous exosomes from mice with acetaminophen-induced liver injury promote toxicity in the recipient hepatocytes and mice

**DOI:** 10.1038/s41598-018-34309-7

**Published:** 2018-10-30

**Authors:** Young-Eun Cho, Wonhyo Seo, Do-Kyun Kim, Pyong-Gon Moon, Sang-Hyun Kim, Byung-Heon Lee, Byoung-Joon Song, Moon-Chang Baek

**Affiliations:** 1 Department of Molecular Medicine, CMRI, School of Medicine, Kyungpook National University, Daegu, 41944 Republic of Korea; 2 Section of Molecular Pharmacology and Toxicology, Laboratory of Membrane Biochemistry and Biophysics, National Institute on Alcohol Abuse and Alcoholism (NIAAA), Bethesda, MD 20892 USA; 3 Laboratory of Liver Diseases, National Institute on Alcohol Abuse and Alcoholism (NIAAA), Bethesda, MD 20892 USA; 4 Mast Cell Biology Section, Laboratory of Allergic Diseases, National Institute of Allergy and Infectious Diseases (NIAID), National Institutes of Health, Bethesda, MD 20892 USA; 5 Department of Pharmacology, School of Medicine, Kyungpook National University, Daegu, 41944 Republic of Korea; 6 Department of Biochemistry and Cell Biology, School of Medicine, Kyungpook National University, Daegu, 41944 Republic of Korea

## Abstract

Exosomes are small extracellular membrane vesicles released from endosomes of various cells and could be found in most body fluids. The main functions of exosomes have been recognized as important mediators of intercellular communication and as potential biomarkers of various disease states. This study investigated whether exogenous exosomes from mice with acetaminophen (APAP)-induced liver injury can damage the recipient hepatic cells or promote hepatotoxicity in mice. We observed that exogenous exosomes derived from APAP-exposed mice were internalized into the primary mouse hepatocytes or HepG2 hepatoma cells and significantly decreased the viability of these recipient cells. They also elevated mRNA transcripts and proteins associated with the cell death signaling pathways in primary hepatocytes or HepG2 cells via exosomes-to-cell communications. In addition, confocal microscopy of *ex vivo* liver section showed that exogenously added exosomes were accumulated in recipient hepatocytes. Furthermore, plasma reactive oxygen species and hepatic TNF-α/IL-1β production were elevated in APAP-exosomes recipient mice compared to control-exosomes recipient mice. The levels of apoptosis-related proteins such as phospho-JNK/JNK, Bax, and cleaved caspase-3 were increased in mouse liver received APAP-exosomes. These results demonstrate that exogenous exosomes from APAP-exposed mice with acute liver injury are functional and stimulate cell death or toxicity of the recipient hepatocytes and mice.

## Introduction

Acetaminophen (APAP) is a widely-used analgesic and antipyretic drug with few side effects when used in therapeutic doses^1^. Although APAP is safe at therapeutic doses, its overdose can cause necrotic hepatic injury in the centrilobular regions and death following acute liver failure^2^. In fact, APAP overdose is a leading cause of drug-induced liver injury (DILI) and increasingly recognized as a significant public health problem^3^, especially in the presence of alcohol (ethanol) drinking^4,5^.

The mechanisms of APAP-mediated hepatotoxic effects are relatively well-established and have been extensively reviewed^6–8^. APAP can stimulate apoptotic or necrotic death pathway as demonstrated in *in vivo* and *in vitro* models^9–11^. The main mechanisms of APAP-induced liver injury can be ascribed to both covalent modifications of various protein targets followed by mitochondrial dysfunction and stimulation of the oxidative stress-mediated cell death pathways^6,7^. For instance, APAP metabolism is known to produce reactive oxygen/nitrogen species (ROS/RNS) and toxic metabolites including *N*-acetyl-*p*-benzoquinone imine (NAPQI), which can bind many cellular proteins and alter their cellular functions^12^. In addition, APAP can stimulate hepatotoxicity by activating the c-Jun *N*-terminal protein kinase (JNK)-related cell death pathway and protein modifications, leading mitochondrial dysfunction and cellular injury^13–15^. In fact, JNK-dependent mitochondria-dependent cell death has been well-established in APAP-induced liver injury^16,17^. Besides, APAP overdose triggers the transcriptional activation of pro-inflammatory factors, such as TNF-α, IL-1β and others in macrophages^18^.

Extracellular vesicles (EVs) are classified into exosomes, macrovesicles, and apoptotic bodies based on their cellular biogenesis and sizes^19^. Exosomes are small membrane vesicles (40–150 nm diameter) that could be secreted from many types of cell and exist in various body fluids such as blood, urine and saliva^20^. Generally, exosomes contain common membrane marker proteins, cell-type specific proteins and nucleic acids, including mRNAs, microRNA (miRNA), other non-coding RNAs and mitoDNAs^21^. One of the major functions of exosomes is to promote intercellular communications in various pathogenic processes such as cancer, cardiovascular disease, diabetes and aging^22,23^. Exosomes are known to be released into the circulation when the parental donor cells in different tissues and organs are exposed to a variety of environmental stimuli, including drugs and toxic agents^21^. In cancer field, exosomes derived from cancer cells can trigger metastasis and promote angiogenesis in recipient cells^24,25^. However, it is poorly understood whether exogenous exosomes derived from mice with APAP-mediated DILI can stimulate cellular toxicity in recipient cells or naïve animals.

Based on diverse biological functions of exosomes, we hypothesized that exosomes derived from APAP-exposed mouse liver can promote cellular toxicity and/or activate the apoptosis signals in recipient cells or mice. Therefore, this work investigated to evaluate whether exogenous exosomes isolated from mice with APAP-induced liver injury can interact with other cells and then increase the oxidative stress with stimulation of the apoptosis signaling pathway, resulting in subsequent damage of the recipient cells and mice.

## Results

### APAP induced liver damage

**Figure 1:**
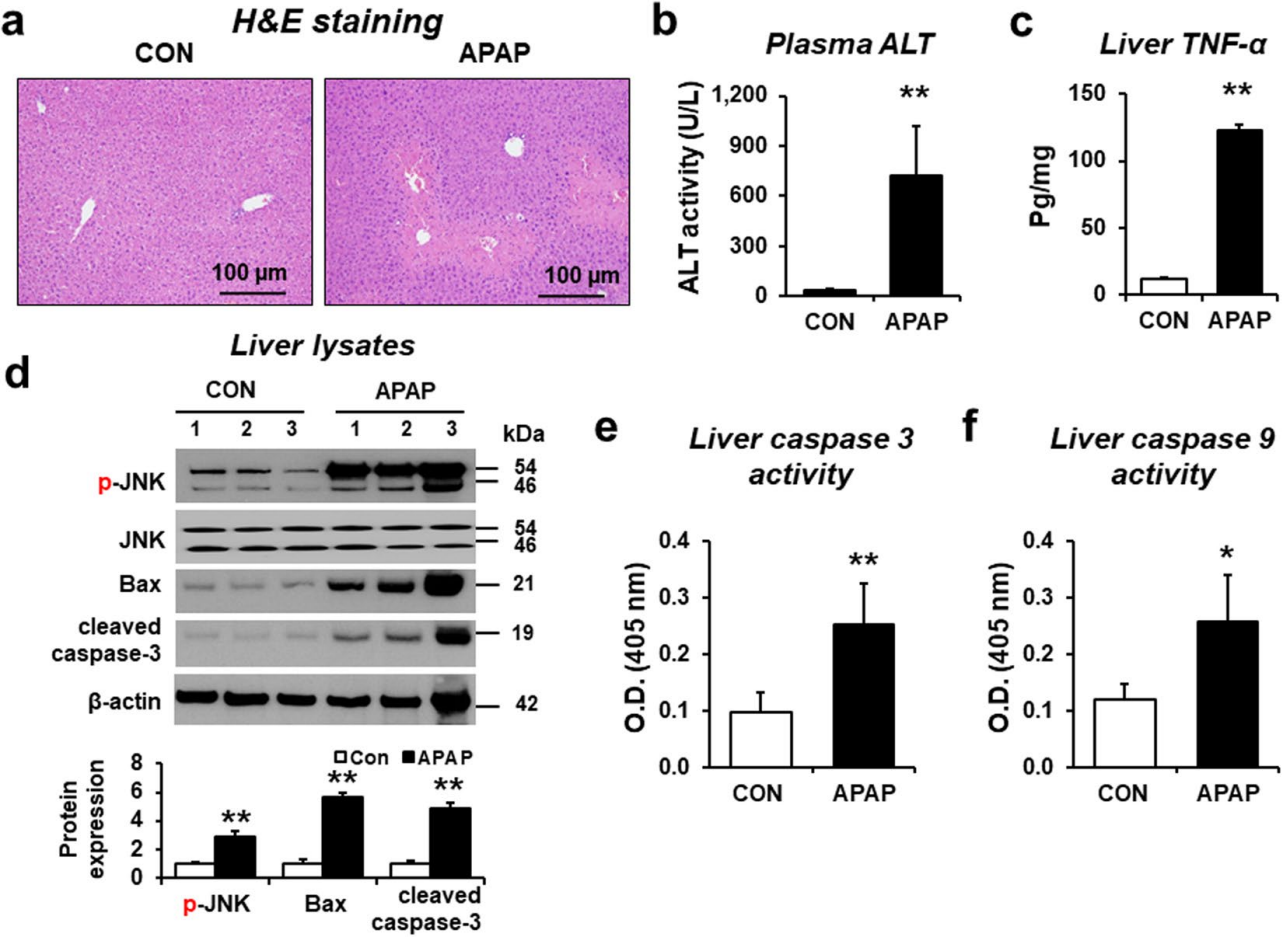
Validation of APAP-induced liver injury in mice and properties of exosomes. (**a**) Representative images of hematoxylin and eosin (H&E) staining for formalin-fixed liver sections in wild-type C57BL/6J mice (n = 8/group). Scale bars, 100 µm. The levels of (**b**) plasma ALT, (**c**) hepatic TNF-α protein for the indicated groups (n = 8/sample). (**d**) Immunoblot showing relative expression of p-JNK, JNK, Bax, cleaved caspase-3, and β-actin, used as a loading control, in liver lysates from control or APAP-administrated mice, as indicated (n = 6/sample). Full-length immunoblots are presented in Supplementary Figure 1. (**e**,**f**) Caspase 3 (**e**) and 9 (**f**) activities (n = 8/sample). The data represent mean ± SD. *P < 0.05, **P < 0.01.

Overdose of acetaminophen (APAP) can induce acute hepatotoxicity in humans and rodents, including mice. Histological analyses revealed severe centrilobular necrosis with markedly elevated plasma ALT activity in mice treated with a single intraperitoneal injection of APAP (300 mg/kg) (Fig. 1a and b). Additionally, the amounts of a hepatic cytokine TNF-α were significantly increased in APAP-exposed mice compared to control mice (Fig. 1c). The cytosolic JNK is rapidly activated (phosphorylated) during APAP toxicity in mice. Phosphorylated JNK (p-JNK) then translocates to mitochondria and triggers the mitochondrial permeability transition (MPT) and mitochondria-dependent cell death^16,17^. To confirm the APAP-induced liver injury, we evaluated the levels of p-JNK, proapoptotic marker protein such as Bax, and cleaved (activated) caspase-3 by immunoblot analyses. Our data showed that the levels of p-JNK/JNK, Bax, and cleaved caspase-3 were markedly elevated in the liver from APAP-exposed mice compared to control mice (Fig. 1d and Supplementary Fig. 1). Indeed, both hepatic caspase-3 and caspase-9 activities were significantly increased in APAP-exposed mice compared to vehicle control (Fig. 1e and f, respectively).

### Properties of APAP-derived exosomes

**Figure 2:**
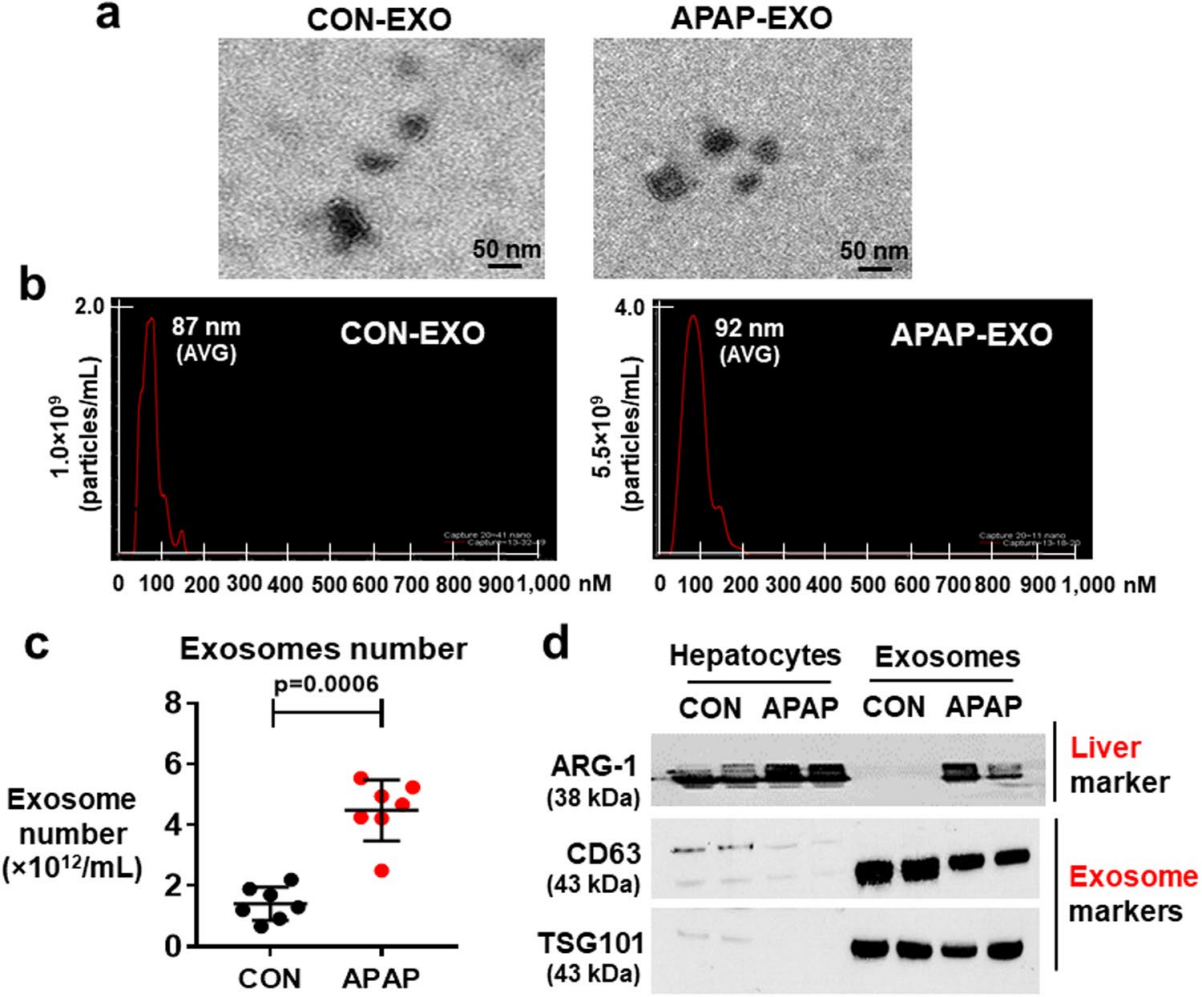
Characterization of exosomes in APAP-induced liver injury. (**a**) A representative electron microscopic image of exosomes prepared from plasma of PBS (CON) or APAP-exposed mice. Scale bar, 50 nm. (**b**) Analysis of the size distribution of exosomes from CON- or APAP-exposed mice with average (avg.) diameters. (**c**) Total number of exosomes in each group was measured by Nanosight. **P < 0.01 (n = 8/sample). (**d**) Representative immunoblot images of liver marker (ARG-1, arginase-1) or exosome markers (i.e., CD63 and TSG101). Full-length immunoblots are presented in Supplementary Figure 2.

To study whether exogenous exosomes from mice with APAP-induced liver damage can promote hepatotoxicity in the recipient cells, APAP-derived exosomes were prepared, as recently described^26,27^. Transmission electron microscopy (TEM) revealed that APAP-derived and control (CON)-derived exosomes displayed round shapes with diameters of approximately 50–100 nm (Fig. 2a). Nanoparticle tracking analysis of exosomes prepared from both control- and APAP-exposed mice showed a majority of small sized particles (Fig. 2b). In addition, the number of exosomes was increased in APAP-exposed mice compared to that of control mice (Fig. 2c). Immunoblot analyses confirmed the presence of comparable amounts of exosomal protein markers CD63 and TSG101 in exosomes isolated from control and APAP-exposed mice (Fig. 2d, lanes 5–8). However, the expressed amounts of both CD63 and TSG101 in the liver lysates of control mice were low and further decreased in APAP-exposed mice (lanes 1–4). We found elevated levels of a liver-specific protein arginase-1 (ARG-1) in exosomes and liver lysates prepared from APAP-exposed mice than those of control mice (Fig. 2d and Supplementary Fig. 2) whereas ARG-1 levels in exosomes isolated from control mice were very low or undetected under our experimental condition. These results showed that APAP-derived exosomes likely contain specific proteins and other components associated with hepatotoxicity, consistent to those described in previous studies^26,27^.

### Uptake and internalization of APAP-derived exosomes by the recipient cells

**Figure 3:**
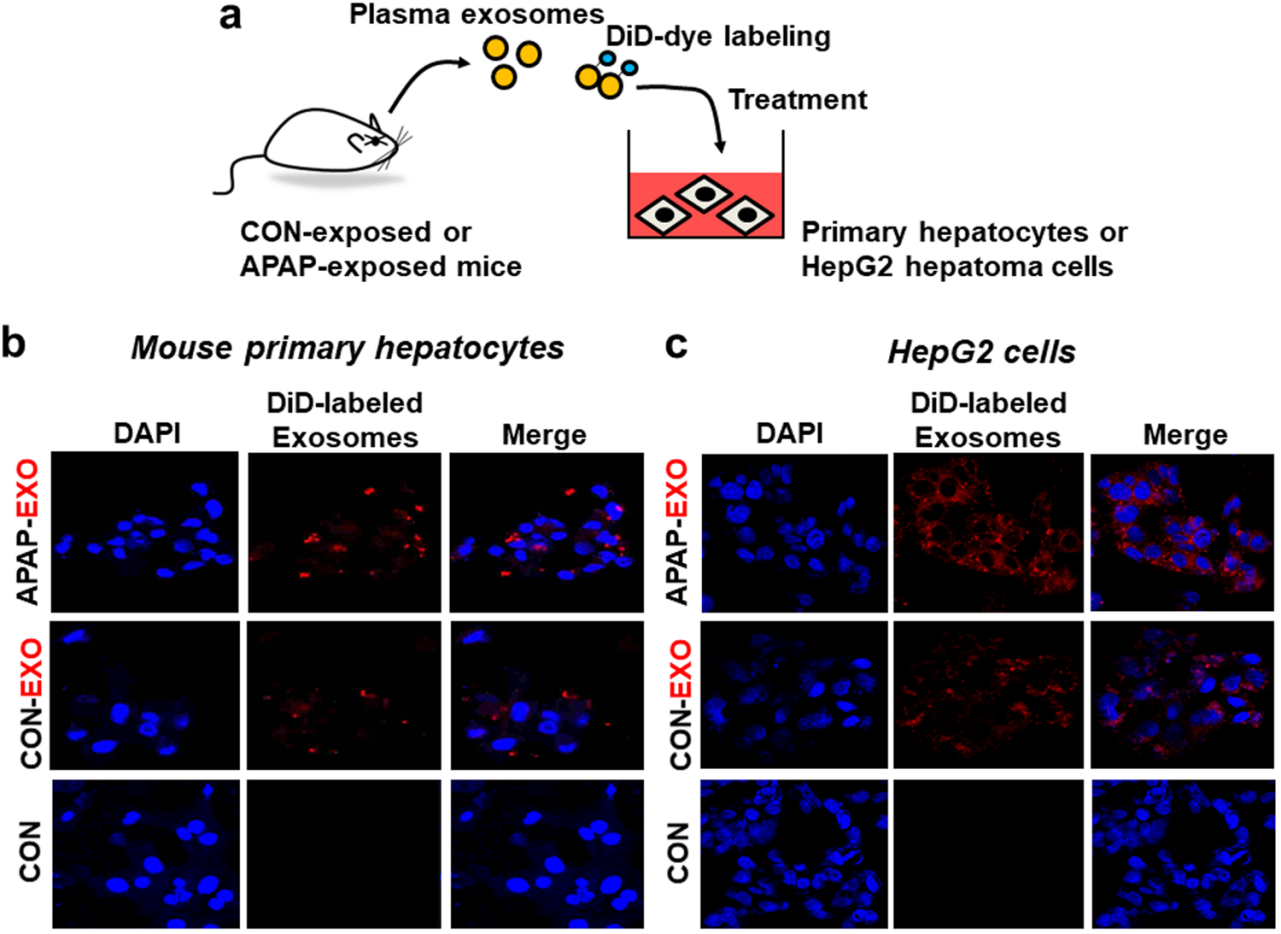
Uptake of APAP-derived exosomes by primary mouse hepatocytes or HepG2 cells. (**a**) Schematic diagram of the experimental procedure. (**b**,**c**) Confocal microscopy images showing internalization of DiD-labeled or unlabeled exosomes from CON- and APAP-exposed mice into primary hepatocytes (**b**) or HepG2 cells (**c**) (n = 4/sample). Cell nuclei were counterstained with DAPI.

To study the potentially toxic effects of exogenous exosomes derived from DILI mice on primary mouse hepatocytes, we isolated exosomes from plasma of APAP-exposed and control mice. The uptakes of DiD-red fluorescence dye labeled exosomes from CON or APAP-exposed mice into primary hepatocytes and HepG2 hepatoma cells were subsequently studied after incubation for up to 24 h (Fig. 3a). Strong signals of the red fluorescence were detected in primary hepatocytes (Fig. 3b) and HepG2 cells (Fig. 3c) after the incubation with DiD-labeled CON or APAP-derived exosomes whereas no fluorescence signals were detected when unlabeled exosomes were incubated. These results suggest that sufficient amounts of DiD-labeled exogenous exosomes were internalized into the recipient cells.

### Elevated expression of the mRNA transcripts associated with cellular toxicity by APAP-derived exosomes

**Figure 4:**
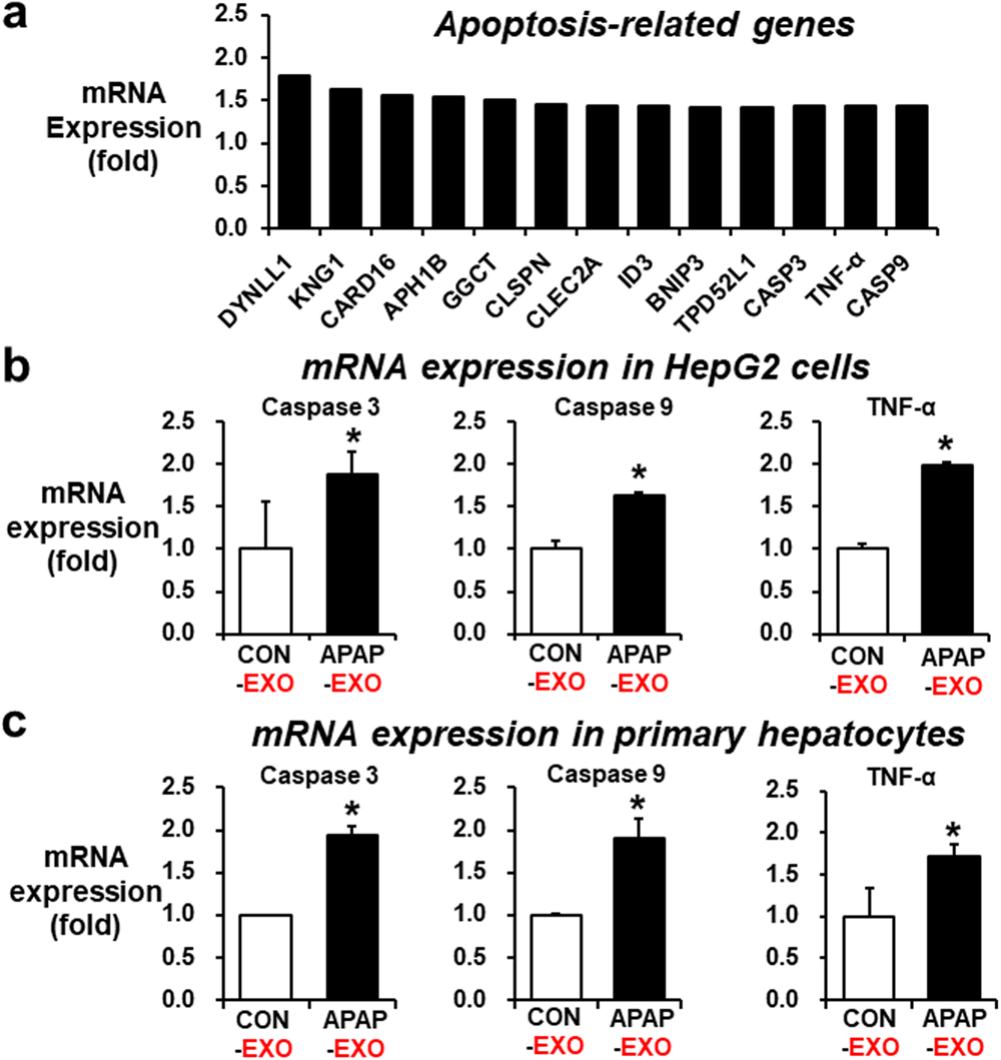
Upregulation of apoptosis marker gene transcripts in HepG2 cells and primary hepatocytes by APAP-derived exosomes. (**a**) 13 mRNA transcripts were upregulated by > 1.5-fold in HepG2 cells treated with APAP-derived exosomes compared with untreated cells (n = 4/sample). (**b**,**c**) Relative expression of *caspase-3, caspase-9*, and *TNF-α* mRNA transcripts in HepG2 cells (**b**) or primary hepatocytes (**c**) after 24 h incubation with APAP-derived exosomes (n = 8/sample). Real-time PCR analysis, determined by the comparative Ct method and normalized using the values of control set at 1, indicating significant differences between exosome-treated cells and untreated groups. *P < 0.05.

To further investigate the potential effects of APAP-derived exosomes on gene expression in hepatic cells, we performed a microarray analysis for more than 30,000 transcripts. The mRNA expression profiles of HepG2 cells treated with 30 µg/mL of APAP-derived exosomes and untreated HepG2 cells (control) were compared by scatter plot analysis. Forty-three mRNA transcripts were unregulated greater than 1.5-fold while 39 mRNA transcripts were down-regulated by <1.5-fold in HepG2 cells after treatment with APAP-derived exosomes. To predict the potential implications of APAP-derived exosomes, Ingenuity Pathway Analysis (IPA) software was used to investigate the functional roles of the differentially expressed genes detected by microarray analysis. Molecular and cellular functions analysis predicted that the upregulated genes are related to cell death and survival, cellular development, cellular growth and proliferation, cell to cell signaling and interaction, cell cycle (Supplementary Fig. 3a). In addition, Genecard analysis strongly predicted that the upregulated genes are associated with oxidative stress, inflammation, and cytokine pathways (Supplementary Fig. 3b). For instance, mRNA transcripts for *DYNLL1* (Dynein light chain 1), *KNG1* (Kininogen 1), *CARD16* (Caspase Recruitment Domain Family Member 16), *APH1B* (Aph-1 Homolog B, Gamma-Secretase Subunit), *GGCT* (Gamma-Glutamylcyclotransferase), *CLSPN* (Claspin), *CLEC2A* (C-Type Lectin Domain Family 2 Member A), *ID3* (Iterative Dichotomiser 3), *BNIP3* (BCL2 Interacting Protein 3), *TPD52L1* (Tumor Protein D52-Like 1), *CASP3* (Caspase-3), TNF-α (Tumor necrosis factor-α), and *CASP9* (Caspase-9) were upregulated in HepG2 cells following treatment with APAP-derived exosomes (APAP-EXO), compared to control-derived exosomes (CON-EXO) (Fig. 4a). Upregulation of *CASP3, CASP9*, and *TNF-α* mRNA transcripts in HepG2 cells or mouse primary hepatocytes exposed to APAP-EXO were validated by real-time PCR analysis (Fig. 4b and c, respectively). Analysis of the molecules altered by the treatment with APAP-EXO revealed significant interacting gene networks related to ‘Cell Death and Survival’, with 25 focus molecules extracted from the differentially expressed genes (Supplementary Fig. 4). All these results strongly suggest that APAP-EXO could activate the cell death signals or apoptosis of the recipient hepatocytes or hepatoma cells.

### Cellular toxicity of the recipient cells by APAP-derived exosomes

**Figure 5:**
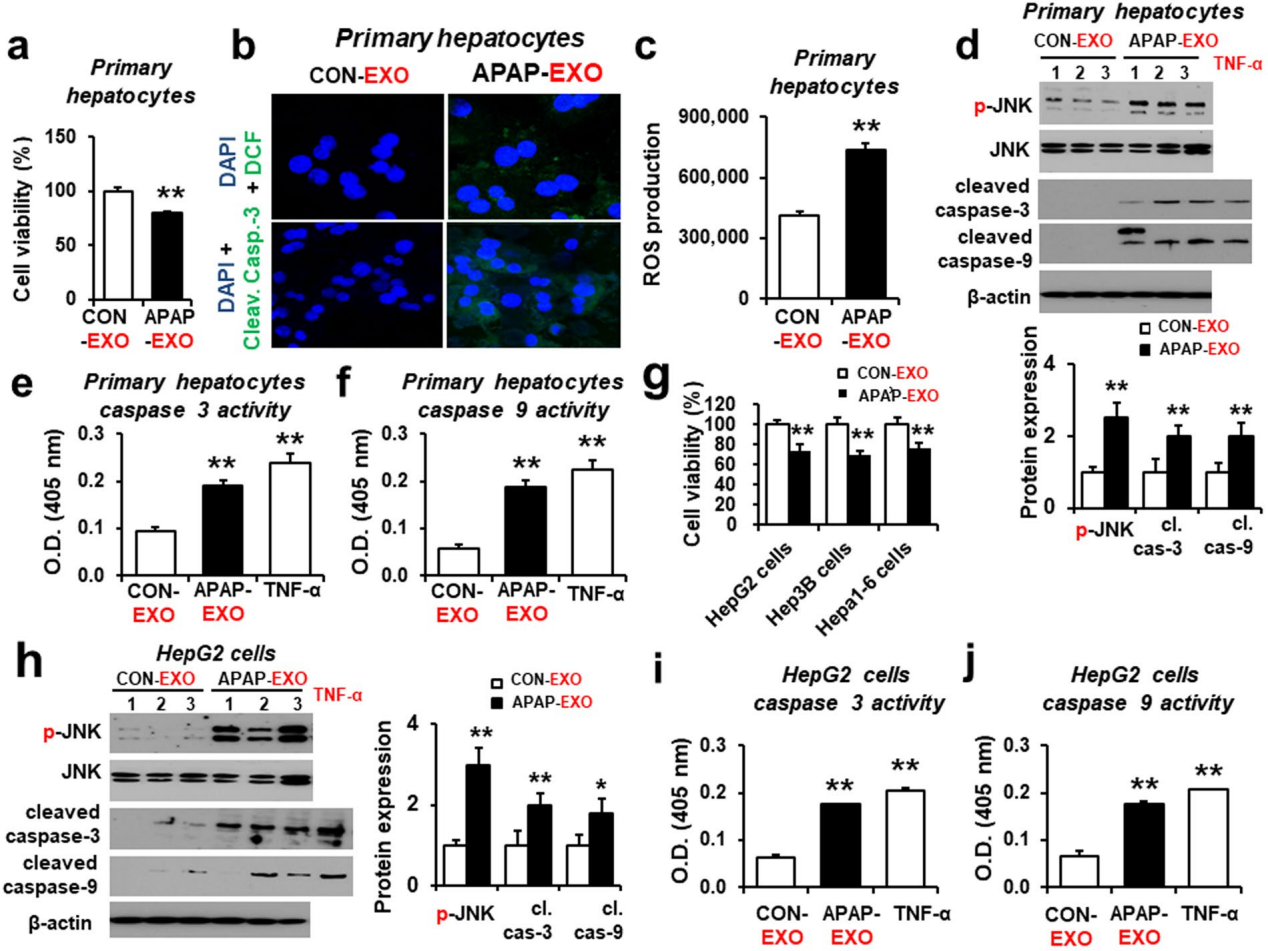
Stimulation of hepatocyte damage by APAP-derived exosomes. (**a**) Cell viability in primary hepatocytes treated with APAP-derived exosomes compared with CON-derived exosomes (n = 8/sample). (**b**) Confocal image showing relative ROS production (top) or cleaved (activated) caspase-3 (bottom) in primary hepatocytes treated with exogenous APAP-derived or CON-derived exosomes for 24 h (n = 8/sample). Primary hepatocytes were counterstained with DAPI to stain the cell nuclei. (**c**) The rate of ROS production in primary hepatocytes after treatment with APAP-derived or CON-derived exosomes (n = 8/sample). (**d**) Immunoblot showing relative expression of p-JNK, JNK, cleaved caspase-3, cleaved caspase-9 and a loading control β-actin in primary hepatocytes treated with APAP-derived or CON-derived exosomes for 24 h (n = 6/sample). Full-length immunoblots are presented in Supplementary Figure 5. (**e** and **f**) Caspase 3 and 9 activities (n = 8/sample). (**g**) Cell viability changes in HepG2, Hep3B, and Hepa1-6 cells treated with APAP-derived exosomes compared with CON-derived exosomes (n = 8/sample). *P < 0.05. (**h**) Immunoblot showing relative expression of p-JNK, JNK, cleaved caspase-3, cleaved caspase-9 and β-actin in HepG2 cells treated with APAP-derived or CON-derived exosomes for 24 h (n = 6/sample). Full-length immunoblots are presented in Supplementary Figure 6. (**i**,**j**) Caspase 3 (**i**) and 9 (**j**) activities (n = 8/sample).

Exosomes have been shown to transfer information between donor and recipient cells and modulate the function of the latter^28^. To examine the effects of exogenous exosomes, the mouse primary hepatocytes were treated with 30 µg/mL exosomes from APAP-exposed mice or control for 24 h followed by cell viability measurements. Viability of the mouse primary hepatocytes was significantly decreased (by ~20%) after treatment with APAP-EXO, compared to the CON-EXO (Fig. 5a). Confocal image data showed that apoptosis probed with cleaved caspase-3 (Fig. 5b) and ROS (DCFH-DA) production (Fig. 5c) were significantly increased in primary hepatocytes exposed to APAP-EXO compared to CON-EXO. Consistently, APAP-EXO elevated the levels of the activated p-JNK/JNK, cleaved caspase-3, and cleaved caspase-9, indicating that APAP-derived exosomes stimulated the cell death signaling pathway in primary hepatocytes (Fig. 5d and Supplementary Fig. 5). TNF-α has also been implicated to stimulate hepatic injury through induction of cellular apoptosis^29^. Our data also showed that TNF-α exposed liver cells under serum starved conditions seem to behave in a similar manner to actinomycin D treatment. Indeed, caspase-3 and caspase-9 activities were significantly elevated in primary hepatocytes exposed to APAP-EXO or TNF-α, compared to CON-EXO (Fig. 5e and f). Similar results of decreased cell viability were also observed in three hepatoma cells (i.e., HepG2, Hep3B, and Hepa1–6 cells) after treatment with APAP-derived exosomes (Fig. 5g). Furthermore, the levels of p-JNK/JNK, cleaved caspase-3, and cleaved caspase-9 were significantly increased in HepG2 cells by APAP-EXO (Fig. 5h and Supplementary Fig. 6). In addition, caspase-3 and caspase-9 activities were significantly elevated in primary hepatocytes exposed to APAP-EXO or TNF-α, compared to CON-EXO (Fig. 5i and j). These results suggest that exosomes derived from APAP-exposed mice stimulated the JNK-mediated cell death signal^30^ in primary hepatocytes and various hepatoma cells, resulting in decreased hepatocyte proliferation.

### Time-dependent effects of APAP-derived exosomes on cell death rates of primary hepatocytes

**Figure 6:**
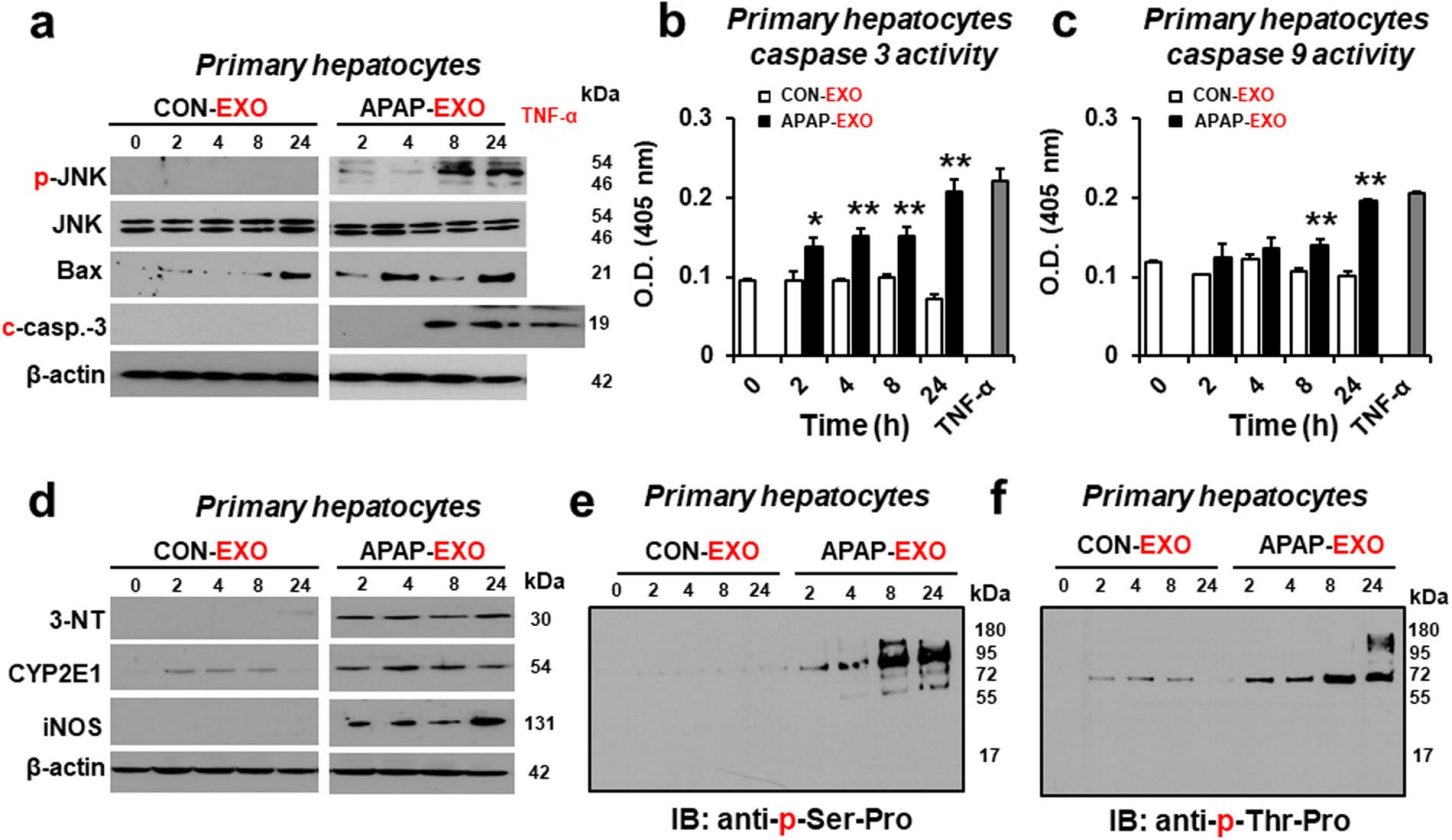
Time-dependent increases in apoptosis-related, nitroxidative stress marker and phosphorylated proteins following exposure to APAP-derived exosomes. (**a**) Immunoblot showing relative amounts of p-JNK/JNK, Bax, cleaved caspase-3 and β-actin, a loading control (n = 4/sample). (**b** and **b**) Caspase 3 and 9 activities (n = 8/sample). (**d**) Immunoblot showing relative amounts of nitroxidative stress markers, 3-NT, CYP2E1, and iNOS compared to β-actin and (**e**,**f**) phosphoproteins containing p-serine-proline (**e**) or p-threonine-proline (**f**) in primary hepatocytes treated with APAP-derived or CON-derived exosomes for 0, 2, 4, 8, and 24 h, as indicated (n = 4/sample). Full-length immunoblots are presented in Supplementary Figure 7.

To determine the time-dependent effects of APAP-EXO on cell death, mouse primary hepatocytes were treated with 30 µg/mL exosomes prepared from APAP-exposed mice or control for 0, 2, 4, 8, and 24 h. Immunoblot results showed that the amounts of p-JNK/JNK, Bax and cleaved caspase-3 in mouse primary hepatocytes were markedly elevated by APAP- EXO in a time-dependent manner where greater effects were observed after longer exposure times (Fig. 6a and Supplementary Fig. 7). Indeed, caspase-3 and caspase-9 activities were significantly elevated in mouse primary hepatocytes exposed to APAP-EXO in a time-dependent manner (Fig. 6b and c).

In addition, nitrative stress marker proteins, such as iNOS and nitrated proteins detected by anti-3-NT antibody, were also elevated in mouse primary hepatocytes after treatment with APAP-EXO in a time-dependent manner (Fig. 6d and Supplementary Fig. 7). However, the levels of CYP2E1, which is also known to elevate oxidative/nitrative stress^13,14^, in the recipient hepatocytes did not seem to be altered following incubation with exogenous APAP- EXO.

**Figure 7:**
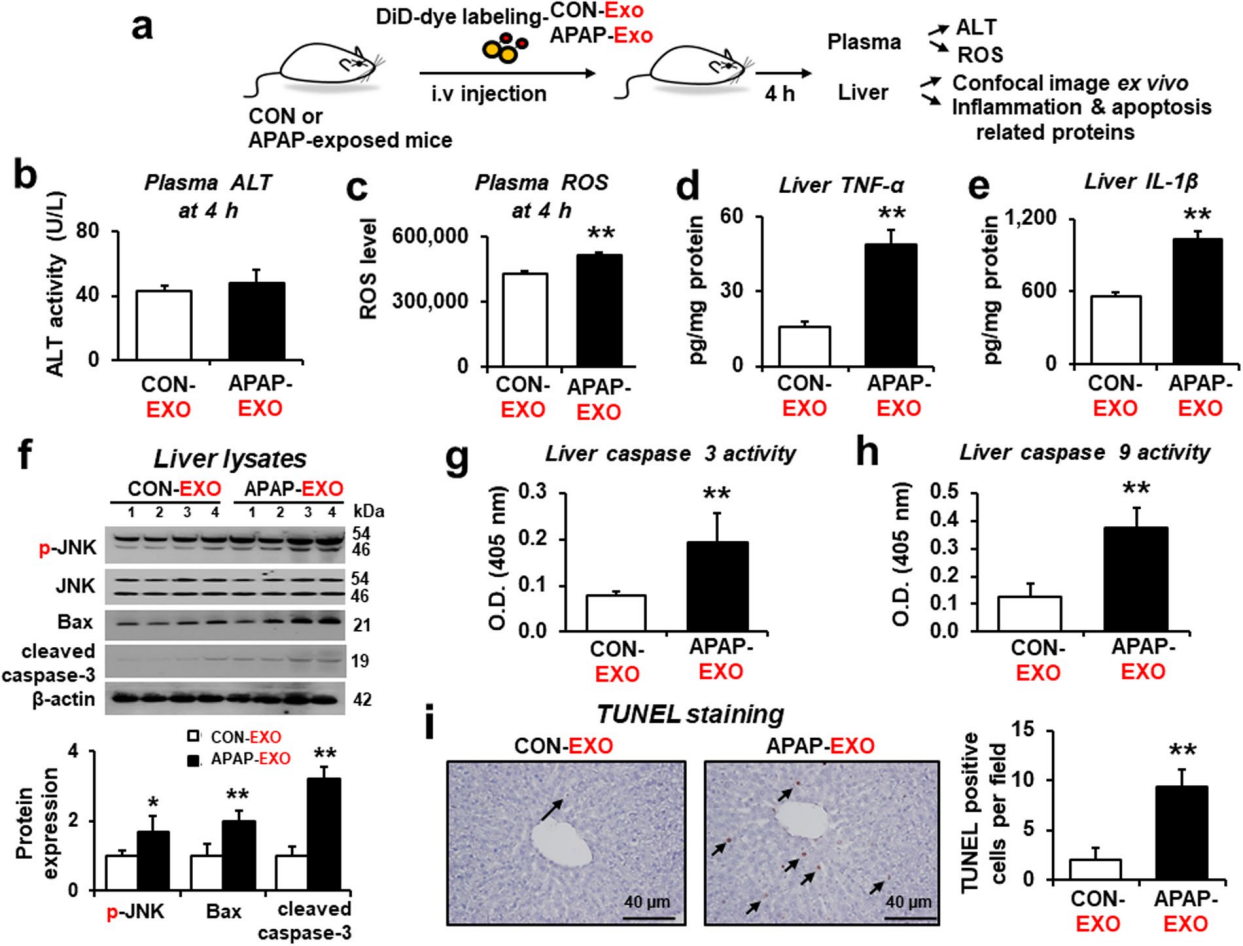
Stimulation of hepatotoxicity in recipient mice by APAP-derived exosomes. (**a**) Schematic diagram of the experimental procedure for the intravenous injection of DiD-labeled exosomes into recipient mice and subsequent analyses. The levels of (**b**) plasma ALT, (**c**) plasma ROS, (**d**) hepatic TNF-α, and (**e**) hepatic IL-1β in recipient mice after treatment with APAP-derived or CON-derived exosomes (n = 5/sample). (**f**) Immunoblot showing relative levels of p-JNK, Bax, cleaved caspase-3 and β-actin in recipient mice after exposure to APAP-derived or CON-derived exosomes (n = 4/sample). (**g** and **h**) Caspase 3 and 9 activities (n = 5/sample). Full-length immunoblots are presented in Supplementary Figure 9. (**i**) Representative TUNEL staining for apoptotic hepatocytes (arrows) (n = 5/sample).

Based on elevated levels of p-JNK after treatment with APAP-EXO (Figs 5 and 7a), we determined the levels of p-JNK target phosphoproteins^30–32^ in the recipient hepatocytes by using anti-p-Ser-Pro or p-Thr-Pro antibody. The amounts of phosphoproteins containing p-Ser-Pro or p-Thr-Pro residues were markedly increased in a time-dependent manner after exposure to APAP-EXO (Fig. 6e and f, respectively). For instance, the amounts of p-Ser-Pro-containing proteins were markedly elevated at 8 h or 24 h while virtually no changes were observed after exposure to CON- EXO. These results suggest that APAP-derived exosomes could induce cell death of the recipient mouse primary hepatocytes through phosphorylation^30–32^ and nitration^13,14^ of many critical proteins^13,14^.

### Increased hepatic cell death signals in the recipient mice exposed to APAP-derived exosomes

We further determined the cellular distribution of the exogenous exosomes within the liver by conducting confocal microscopy to collect the fluorescent signals from the *ex vivo* liver section at 4 h after intravenous injection of DiD-labeled exosomes. Confocal image results revealed intensive fluorescent signals in hepatocytes (Supplementary Fig. 8), indicating that hepatocytes are likely the major cells where exogenously added exosomes accumulated.

We then tested the biological effects of exogenous APAP- EXO on hepatotoxicity in the recipient mice (Fig. 7a). Plasma ALT levels were unchanged 4 h after i.v. administration of APAP-EXO compared to CON-EXO (Fig. 7b). Interestingly, plasma ROS production was significantly elevated in recipient mice after injection of APAP-EXO compared to mice received CON-EXO (Fig. 7c). Additionally, hepatic TNF-α and IL-1β proteins were significantly increased in recipient mice treated with APAP-EXO (Fig. 7d and e, respectively). Immunoblot analysis showed significantly elevated hepatic p-JNK/JNK, Bax, and cleaved caspase-3 proteins in recipient mice exposed to APAP-EXO compared to those with CON-EXO (Fig. 7f and Supplementary Fig. 9). Additionally, hepatic caspase-3 and caspase-9 activities were significantly elevated in the recipient mice exposed to APAP-EXO compared to those with CON-EXO (Fig. 7g and h, respectively). TUNEL analysis showed markedly elevated apoptosis of hepatocytes in the recipient mouse liver after administration of APAP- EXO (Fig. 7i). All these results clearly demonstrate that exosomes prepared from APAP-exposed mice could elevate ROS production, inflammatory and/or apoptosis-related marker proteins, leading to increased hepatotoxicity in the recipient mice.

### Decreased APAP-induced cell death and TNF-α production by inhibition of exosomes secretion

We have recently reported that exogenous EVs from alcoholic hepatitis patients or alcohol-exposed mice could cause cellular toxicity in the recipient hepatocytes and that the hepatotoxicity depended on the levels of exosome secretion^33^. To further confirm whether inhibition of exosomes secretion can prevent APAP-induced hepatotoxicity, we pretreated the mouse primary hepatocytes with GW4869 or dimethyl amiloride (DMA) as an EV secretion inhibitor. Pretreatment with GW4869 or DMA significantly decreased the TNF-α production and number of exosomes in cell culture supernatants released from the primary hepatocytes after exposure to APAP (Supplementary Fig. 10a and b). Additionally, pretreatment with GW4869 or DMA markedly increased the viability of APAP-exposed hepatocytes (Supplementary Fig. 10c). Our results showed that the elevated APAP-induced hepatotoxicity could be significantly prevented by inhibition of exosomes secretion.

## Discussion

We and other laboratories have recently reported that the levels of liver-specific proteins and/or miRNAs in circulating plasma exosomes were significantly increased in animals and humans exposed to hepatotoxic agents such as APAP and alcohol^26,27,33,34^. In addition, these exosomal miRNAs and proteins were increased through oxidative and endoplasmic reticulum (ER) stress since treatments with an antioxidant or ER stress inhibitor significantly suppressed the elevated number of exosomes and the amounts of their contents by hepatotoxic agents^33^. These exosomal miRNAs and proteins such as CYP2E1 can be used the potential biomarkers for drug- or alcohol-induced liver injury^26,27,33^. In this study, we investigated whether exogenous exosomes containing miRNAs and proteins can regulate the fates of recipient primary hepatocytes or hepatoma cells as well as tissue distribution with hepatic or renal toxicity in living mice. This study first shows that exosomes, released from mice with APAP-induced DILI, exhibited the functional capacity to communicate with the recipient cells to stimulate the cell death signal with increased phosphorylation and nitrative stress, resulting in decreased cell viability of mouse primary hepatocytes and HepG2 hepatoma cells. In addition, our microarray analysis with HepG2 hepatoma cells revealed that the APAP-derived exosomes upregulated various mRNA transcripts and proteins associated with cell death or apoptosis mechanism and that upregulation of some of these transcripts was confirmed in primary hepatocytes and HepG2 hepatoma cells. The proteins or other components such as miRNAs in the exosomes from mice with APAP-induced liver injury were internalized into the recipient cells and stimulated cellular toxicity in primary hepatocytes and HepG2 cells through increased production of ROS and RNS reflected by elevated iNOS and nitrated proteins. These results suggest that cellular toxicities observed in the recipient cells could have, at least partly, resulted from elevated nitroxidative stress, which activates the stress-activated p-JNK and other protein kinases involved in cell death signaling^30–32^.

Exosomes harvested from different cells or tissues under various pathophysiological conditions or after exposure to an exogenous agent have been shown to exert various biological effects, depending on the type of stimulation and the host target cells^35^. Lotvall and colleagues reported that exosomes can influence the response of recipient cells to oxidative stress stimulus by transferring RNAs from one type of cells to another^36^. Other investigators reported that circulating exosomes released from cancer cells can communicate with recipient cells, resulting in docetaxel-resistance^37^. Feng and coworkers also showed the role of exogenous exosomes in the regulation of the sensitivity and resistance of A549 cells to cisplatin^38^. In addition, lipid-induced DR5 ligand-independent activation results in the release of extracellular vesicles (EVs) from hepatocytes and these exosomes were shown to induce an inflammatory macrophage phenotype^39^. Furthermore, exosomes derived from hepatocytes play a beneficial role in liver repair and regeneration after ischemia/reperfusion injury^40^. However, the effects of APAP-derived exosomes on the cell and organ toxicity in the recipient cells or living mice have not been systematically studied. The current study would add to the list of biological functions of exogenously added exosomes by showing that APAP-derived exosomes promote the cell death signaling pathway in recipient hepatic cells and living mice through increased nitroxidative stress, protein modifications and cell death signaling pathway.

In this study, cellular uptake and distribution of exogenous exosomes were also investigated by confocal imaging analysis by using red fluorescent dye DiD-labeled APAP-derived exosomes in liver cells *in vitro and ex vivo* systems. We observed the elevated red-fluorescence signals in mouse primary hepatocytes and liver in living mice after exposure to DiD-labeled APAP-derived exosomes. These data suggest exogenously administered exosomes were internalized into liver cells and stimulated hepatotoxicity or liver injury. Our results showed that APAP-derived exosomes activated the hepatic cell death pathways as analyzed by top significant Ingenuity Pathway Analysis (IPA) gene network using the differential expressed genes. The bioinformatics predictions of activated cell death pathways were supported by the increased levels of cleaved caspase-3, the most important apoptosis-associated protein and hepatocellular apoptosis as the first step^41^ and cleaved caspase-9 with elevated cytosolic cytochrome c released from mitochondria^42^.

APAP-mediated liver injury has been extensively studied as a prototype model of DILI. Many laboratories and we have reported different mechanisms of liver injury. These mechanisms include: protein-adduct formation with NAPQI^12^, a major reactive metabolite of APAP, activation of JNK-mediated cell death signaling pathway^15–17^, CYP2E1-dependent protein nitration^14,43^ followed by mitochondrial dysfunction and apoptosis, etc. APAP can stimulate apoptotic or necrotic death pathway in cells and mice^9–11^. For instance, mice treated with a toxic dose of acetaminophen contained 40% apoptotic and 60% necrotic hepatocytes^44^. However, to our knowledge, the direct or indirect effects of exogenous exosomes from APAP-exposed mice on toxicity of healthy primary hepatocytes and liver in living animals have not been systematically studied. Our current results showed that APAP-derived exosomes significantly promoted death of the hepatocytes or hepatoma cells and liver injury in living mice. The underlying mechanisms of toxicities of APAP-derived exosomes could be due to elevation of cell death associated genes, p-JNK mediated protein phosphorylation and iNOS-nitrative stress-mediated protein nitration, leading to mitochondrial dysfunction, and direct activation of the cell death signaling pathway. In fact, persistent activation of p-JNK along accompanied with elevated phosphoproteins and nitrated proteins were observed as early as 4 h after exposure to exogenous APAP-derived exosomes. It is likely that these covalent protein modifications may play a contributing role in cell death, as reported^13,14,32^. In this case, the level of CYP2E1, which is known to produce ROS and oxidative stress^14,15^, was not elevated by addition of APAP-derived exosomes. These results may suggest a permissive role of CYP2E1 for other proteins/genes to promote damaging effects, as reported^45^. None-the-less, all these early time-dependent cellular changes following administration of exogenous exosomes are likely to contribute to promoting hepatotoxicity in the recipient cells/tissues in an additive/synergistic manner, as recently exemplified^46^. Furthermore, these additional results with the exogenous exosomes from CCl_4_-exposed mice (data not shown) or rodents with alcoholic liver injury or patients with alcoholic hepatitis (data not shown) support all the current results with the APAP-derived exosomes. In this connection, all these results with different hepatotoxic agents (e.g., APAP, CCl_4_, and alcohol)^26,27,33,34^ further suggest that exosomes derived from any liver injury such as nonalcoholic steatohepatitis or viral hepatitis may also negatively affect the physiology of adjacent hepatocytes (or other cells in the liver), although this assumption needs to be experimentally verified.

Recent review articles have suggested many pathophysiological roles of exogenous exosomes. For instance, the exosomal components can be used reliable biomarkers for certain disease states such as breast cancer^47,48^ while exosomes can be used as physiological carriers for better drug delivery to the target cells/tissues with reduced side effects^49,50^. Furthermore, exogenous exosomes can directly exert the beneficial or damaging effects on the recipient cells/tissues, depending on the cell or tissue source and status of the recipient cells, contents of exosomes such as cancer drugs for drug delivery, etc, as recently reviewed^51^. If the damaged recipient cells are used, exogenous exosomes prepared from stem cells are likely to be beneficial in repairing the damage, as exemplified^40^. In contrast, exosomes derived from disease states, as in the case of APAP- or alcohol exposed liver injury^33^, can exert harmful effects on the healthy recipient cells. Although APAP-derived exosomes should contain a variety of components, including APAP or its reactive metabolites, it is unknown which components can actually stimulate the liver toxicity and this area still needs be studied in the future.

In conclusion, in this study we have shown that exosomes prepared from mice with APAP-induced liver injury were internalized and exhibited their functional ability with stimulating cellular toxicity or apoptosis of the recipient cells in culture and living mice. These effects could be resulted from the exchanges of the various exosomal components including proteins, miRNAs, mRNAs and/or metabolites that could be elevated by APAP exposure. This current study shows, for the first time, that the APAP-derived exosomes could be functional to promote the cellular toxicity of the primary hepatocytes or hepatoma cells as well as liver injury in living animals through increased nitroxidative stress, protein modifications and activated cell death signaling pathways.

## Material and Methods

### Antibodies

Antibodies against p-JNK (#9255), JNK (#9252), cleaved caspae-3 (#9661), cleaved caspae-9 (#9509), p-Ser-Pro (#2325), and p-Thr-Pro (#9391) were from Cell Signaling Technology (Beverly, MA) and used at a dilution of 1:1,000 for immunoblot analysis. Antibodies against Bax (sc-20067, diluted to 1:1,000), CD63 (sc-15363, diluted to 1:1,000), TSG 101 (sc-7964, diluted to 1:1,000), and β-actin (sc-47778, diluted to 1:2,000), were from Santa Cruz Biotechnology (Dallas, TX). Antibodies against CYP2E1 (ab28146, diluted to 1:3,000), 3-NT (sc-ab6139, diluted to 1:3,000), and iNOS (ab136918, diluted to 1:1,000), were from Abcam (Cambridge, Mass).

### Animals studies

All animal experimental procedures were carried out by following the National Institutes of Health (NIH) guidelines for small animal experiments and approved by the Kyungpook National University, Daegu, Korea and NIAAA Institutional Animal Care and Use Committee. The animals housed at 20 ± 2 °C with 12 h light/dark cycles and a relative humidity of 50 ± 5% under filtered, pathogen-free air, with food and water available *ad libitum*, and were stabilized for at least 2 weeks prior to each experiment. To prepare a model of DILI, six-month-old male C57BL/6 J mice were exposed to a single injection with saline or 300 mg/kg APAP (intraperitoneally i.p.) for 24 h.

### Exosome isolation

Plasma exosomes were isolated by following the methods, as previously described^26^. Briefly, mouse plasma samples were prepared by centrifugation at 2500 rpm for 10 min and 100 μL of the supernatants were mixed with ExoQuick solution and incubated at 4 °C for 30 min before centrifugation at 1,500 × g for 30 min. After initial precipitation of exosomes with ExoQuick solution, re-pelleting was performed (1,500 *g* for 5 min) three times until the exosomes appeared as a beige or white pellet at the bottom of the vessel. The final pellet was reconstituted in 100 µL of PBS buffer and then stored at 4 °C for up to 7 days or at −20 °C for long-term storage.

### Transmission electron microscopy

For negative staining, isolated exosomes were washed in PBS twice. Briefly, exosomes were mounted on copper grids, fixed by 2.5% glutaraldehyde in cold PBS for 10 min to stabilize the immunoreaction. Exosomes were washed twice in PBS, contrasted by 2% uranyl-oxalate solution at pH 7 for 15 min, embedded by methyl cellulose-UA for 10 min on ice. Images of exosome samples were recorded with an Olympus SIS Veleta CCD camera at a voltage of 80 kV.

### Nanoparticle tracking analysis (NanoSight^TM^)

The number and size of exosomes isolated were measured by nanoparticle tracking analysis (NTA) using a NanoSight NS300 system (NanoSight, Amesbury, UK). The instrument was calibrated with 100 nm polystyrene beads (Thermo-Fisher Scientific, Fremont, CA) before being used. NTA software was used to measure the concentration of nanoparticles (particles/mL) and the size distribution in nm. The Batch Process included in the NTA software was used to integrate the three technical measurements of each sample. The mean vesicle size as well as the concentration of each preparation were obtained from analysis and corrected by the dilution factor when required. Each sample was measured at least 3 times.

### Cell culture

The human hepatoblastoma cell line HepG2 was obtained from the ATCC. HepG2 cells were cultured in DMEM supplemented with 10% FBS and 1% antibiotic-antimycotic solution at 37 °C under 5% CO_2_, as previously described^33^. Primary mouse hepatocytes were freshly isolated from male mice, using a two-step collagenase perfusion procedure, as previously described^33^. Primary hepatocytes were incubated in a humidified incubator under 95% air and 5% CO_2_ at 37 °C for 24 h.

For the experiments with exosomes treatment, cell culture medium was centrifuged at 100,000 *g* overnight to prepare vesicle-depleted (VD) medium by spinning down any preexisting vesicular contents. Mouse primary hepatocytes or HepG2 human hepatoma cells were incubated with exosomes (30 µg of exosomal proteins) in DMEM medium containing 10% FBS and 1% antibiotic-antimycotic solution. After 24 h incubation, cultured cells were collected by centrifugation at 3,000 *g* for various biochemical and immunological analyses.

As a positive control for apoptosis, HepG2 cells were synchronized by serum starvation for at least 12 h before TNF-α treatment. Synchronized HepG2 cells were treated for 24 h with recombinant human TNF-α (50 ng/mL; ab9740) diluted in fresh medium, as reported^52^.

After reaching 80~90% confluence, primary hepatocytes were also synchronized by serum starvation for at least 12 h before TNF-α treatment. The primary hepatocytes were treated for 24 h with recombinant mouse TNF-α (20 ng/mL; ab9642) diluted in fresh medium, as described^53^.

### Labeling of exosomes, immunofluorescence and confocal microscopy

The APAP-derived and control-derived exosomes were labelled using DiD (Thermo-Fisher) red fluorescent dye for membrane labeling, according to the manufacturer’s instructions at 1: 200 dilution. Briefly, exosomes were mixed with 1 mL DiD dye solution and incubated for 5 min. After ultra-centrifugation at 100,000 *g* for 70 min at 4 °C, the DiD-labeled exosome pellets were washed with PBS and centrifuged again at 150,000* g* for 90 min to remove the free, unbound dye. The final resuspended pellets were used as DiD-labeled exosomes.

Mouse primary hepatocytes were initially plated onto chamber slides. The cells were then incubated with the indicated antibodies at 4 °C overnight. Cleaved caspase-3 antibodies, DiD-labeled exosomes, or ROS (DCFH-DA) in cultured cells were detected with Alexa Fluor 488-labeled anti-rabbit secondary antibody (Invitrogen) at 1:300 dilution. For nuclear staining, the cultured cells were incubated with 1 mg/mL 4′,6′-diamino-2-phenylindole (DAPI) for 5 min. The cells were washed and mounted with VECTASHIELD mounting medium. Fluorescent cell images were collected using a confocal microscope (Carl Zeiss).

### Cell viability

Cell viability was determined by cell proliferation assay based on the metabolic reduction of 3-[4,5-dimethylthiazol-2-yl]-2,5-diphenyltetrazolium bromide (MTT). After replacing the media with 1 × phosphate buffered saline (PBS), MTT solution (10 µL of 5 mg/mL in PBS into 0.1 mL final volume) was added to each well, and then incubated at 37 °C for 3 h to allow the occurrence of formazan crystals, which were subsequently dissolved in DMSO. Absorbance of each microtiter plate well was read at 570 nm with a microplate reader (BioTek). Results were relatively expressed to control values specified for each experiment.

### Immunoblot analysis

Part of livers, cultured *cells* or exosomes were lysed with RIPA buffer. Protein concentrations were determined using the BCA Protein Assay Kit (Thermo Fisher Scientific). Equal amounts of protein (a total of 50 µg/lane) from different groups as indicated in the figures were separated by SDS/PAGE and transferred to nitrocellulose membranes for immunoblot analyses. Each indicated target protein recognized by the specific primary antibody. Relative protein images were determined by using HRP-conjugated secondary antibodies and ECL substrates (Thermo Fisher Scientific). The densitometric intensities of the immunoreactive bands were quantified by using ImageJ software (National Institutes of Health).

### Microarray analysis

Total RNA was isolated from HepG2 cells using an RNeasy^®^ Mini Kit (Qiagen) as previously described^54,55^. Global scaling normalization was performed and normalized results were then log-transformed with base. Differentially expressed genes (DEGs) were selected based on a >1.5-fold change and Welch’s t-test (*p < *0.05). Pathway resources were determined by using Ingenuity Pathway Analysis (Qiagen).

### RNA extraction and real-time analysis

Total RNA was extracted from HepG2 hepatoma cells or mouse primary hepatocytes using RNeasy® plus Mini Kit (Qiagen) by following the manufacturer’s recommendations. The concentration of RNA samples was measured by Nanodrop® ND-1000 (Thermo Fisher Scientific). For real-time analysis, cDNA was transcribed from a total of 100 ng of DNase I–treated RNA using the SuperScript III Reverse Transcriptase (Invitrogen) and random primers (Invitrogen). Real-time quantitative PCR amplification reactions were carried out in 7900HT Sequence Detection System from Applied Biosystems in a 20 μl volume. To determine relative mRNA expression, housekeeping gene (β-actin) and apoptosis marker gene with SYBR green I (SYBR Advantage qPCR Premix) were used.

### *In vivo* distribution of exosomes

The DiD-red fluorescent dye (Thermo-Fisher) was used to study distribution of exosomes *in vivo*. APAP-derived or CON-derived exosomes were labeled with a red fluorescent dye DiD (20 µM final concentration in PBS) by incubation for 30 mins, followed by centrifugation at 10,000 *g* for 1 h to remove the unbound dye. Exosomes pellets were suspended in PBS and sterilized by passing 0.22 µM filter. To determine cellular distribution by *ex vivo* imaging analyses, DiD-labeled exosomes from APAP-exposed or control mice were administered into mouse tail vein (C57BL/6J, n = 5/group) via intravenous injection. At 4 h after administration, mouse livers were excised and subjected to confocal microscopy to collect fluorescence from DiD-labeled exosomes.

### Measurements of plasma ROS levels

Total ROS levels in individual plasma and hepatocytes samples were measured using OxiSelect *In Vitro* ROS/RNS Assay Kit (Cell Biolabs, Inc.) following the manufacturer’s protocol. In brief, serum samples were diluted in PBS (1:100), equilibrated at room temperature and then incubated for 15 min with stabilized dichlorodihydrofluorescein diacetate (DCFH-DA). Fluorescence from the DiD-labeled exosomes was measured at 485 and 535 nm excitation and emission wavelengths, respectively, with a plate reader.

### TUNEL assay

The liver sections were fixed overnight in 10% buffered formalin and embedded in paraffin. The ApopTag peroxidase *in situ* apoptosis detection kit (Millipore, Billerica, MA) was used to identify apoptotic hepatocytes by the TUNEL analyses, as recently described^56^.

### Enzyme-linked immunosorbent assay (ELISA)

Equal amounts of the liver lysates prepared from individual mice received APAP-derived or CON-derived exosomes were analyzed by using the respective ELISA kits for TNF-α (Thermo Fisher Scientific) and IL-1β (Thermo Fisher Scientific) by following the manufacturer’s protocols. Duplicate samples from each lysate (n = 5/group) were used for ELISA, which was repeated twice.

### Caspase 3 and 9 activities

Caspase 3 and 9 activities were measured in tissue homogenates or cell lysates using a commercial kit from Abcam (caspase 3: ab39401 and caspase 9: ab65608, respectively) according to the manufacturer’s instructions.

### Statistical analysis and other methods

The data represent average ± SD. For correlation analysis, Pearson’s correlation test was performed in GraphPad Prism software. The nonparametric Mann-Whitney test or two-tailed t test was employed for statistical analysis. *P* < 0.05 was considered statistically significant. The experiments were repeated at least twice, unless otherwise described. Other methods not specifically described here were conducted as same as recently described^26,27,33,46^.

## Electronic supplementary material

Supplementary information
