# Supplementary material for: Exogenous exosomes from mice with acetaminophen-induced liver injury promote toxicity in the recipient hepatocytes and mice
Source: Sci Rep. 2018 Oct 30;8:16070. doi: 10.1038/s41598-018-34309-7 (PMC6207703; doi:10.1038/s41598-018-34309-7)
Supplement: Supplementary file 1 — Supplementary information [file 41598_2018_34309_MOESM1_ESM.pdf]

Supplementary information

Exogenous exosomes from mice with acetaminophen-induced liver injury promote toxicity in the recipient hepatocytes and mice

Young-Eun Cho[1,2], Wonhyo Seo[3], Do-Kyun Kim[4], Pyong-Gon Moon[1], Sang-Hyun Kim[5], Byung-Heon Lee[6], Byoung-Joon Song[2], Moon-Chang Baek[1]*

Table contents

Supplementary Fig. 1 …….. 2

Supplementary Fig. 2 …….. 3

Supplementary Fig. 3 …….. 4

Supplementary Fig. 4 …….. 5

Supplementary Fig. 5 …….. 6

Supplementary Fig. 6 …….. 7

Supplementary Fig. 7 …….. 8

Supplementary Fig. 8 …….. 9

Supplementary Fig. 9 …….. 10

Supplementary Fig. 10 …….. 11

Supplementary Figure

Supplementary Fig. 1.

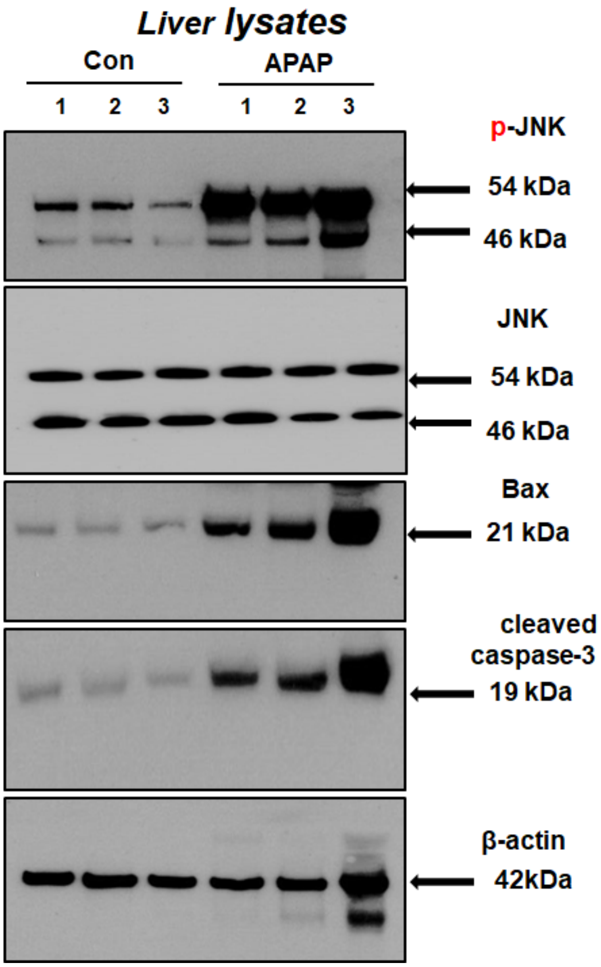

Supplementary Fig. 1. Full-length immunoblots for Fig. 1D.

Supplementary Fig. 2.

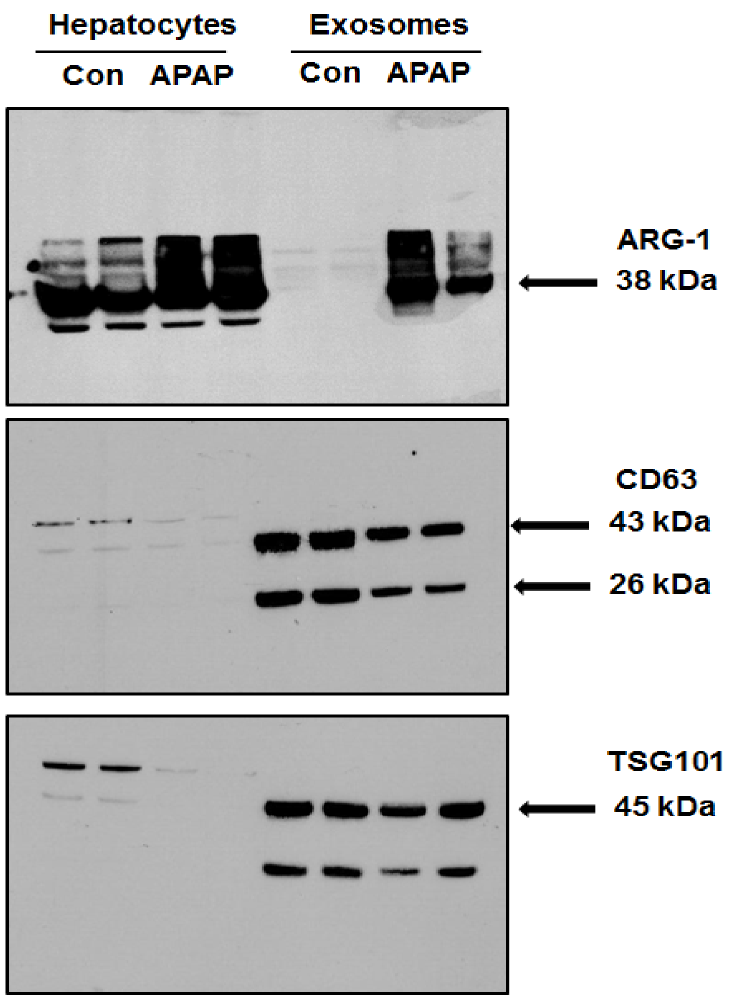

Supplementary Fig. 2. Full-length immunoblots for Fig. 2D.

Supplementary Fig. 3.

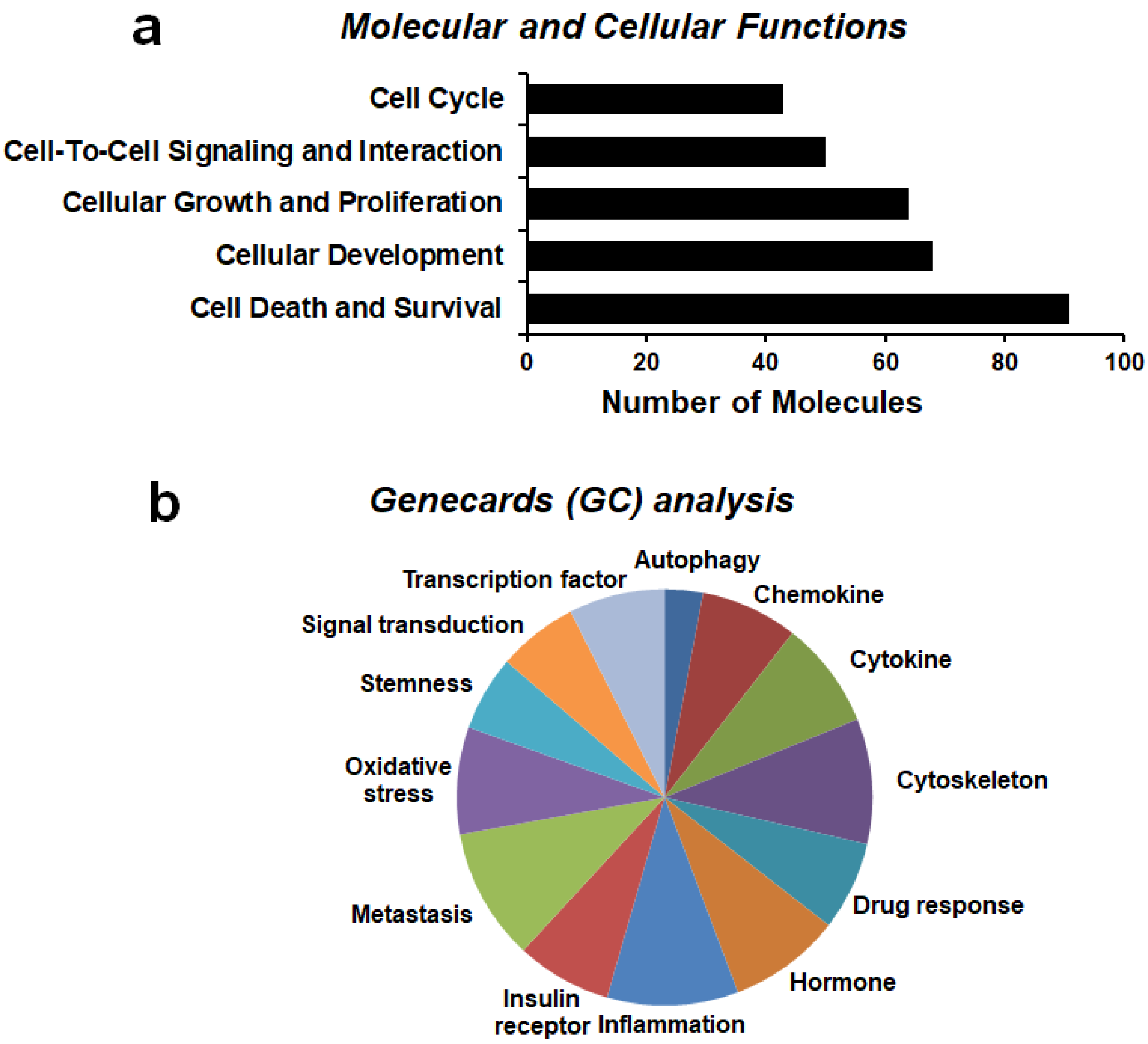

Supplementary Fig. 3. Altered gene expression in HepG2 hepatoma cells following treatment with APAP-derived exosomes. (A) Gene ontology analysis was performed by DAVID program. (B) Genecards analysis was performed by DAVID program.

Supplementary Fig. 4.

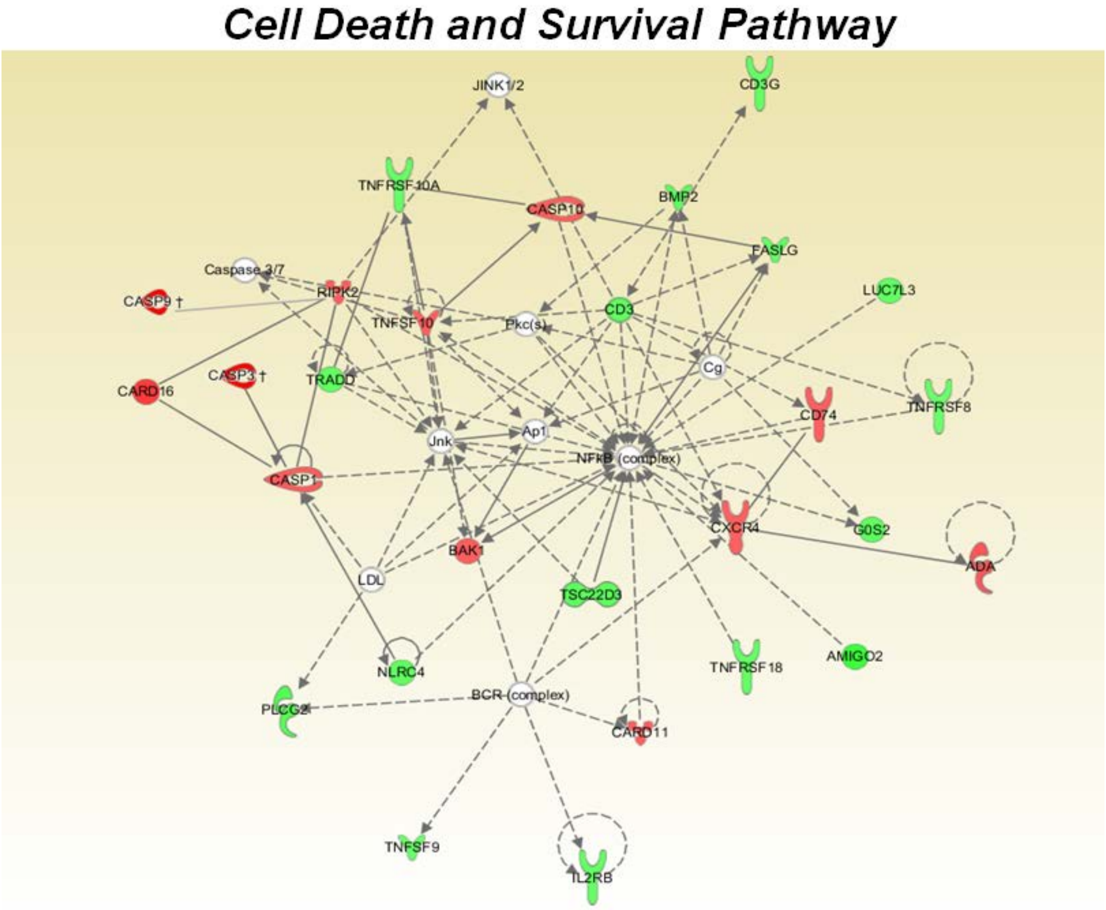

Supplementary Fig. 4. Top significant Ingenuity Pathway Analysis (IPA) gene network using the differential expressed genes in HepG2 hepatoma cells after treatment with APAP-derived exosomes. Data were analyzed through the use of IPA (QIAGEN Inc., https://www.qiagenbioinformatics.com/products/ingenuitypathway-analysis). Solid and dotted lines denote direct and indirect relationships, respectively, between genes/molecules.

Supplementary Fig. 5.

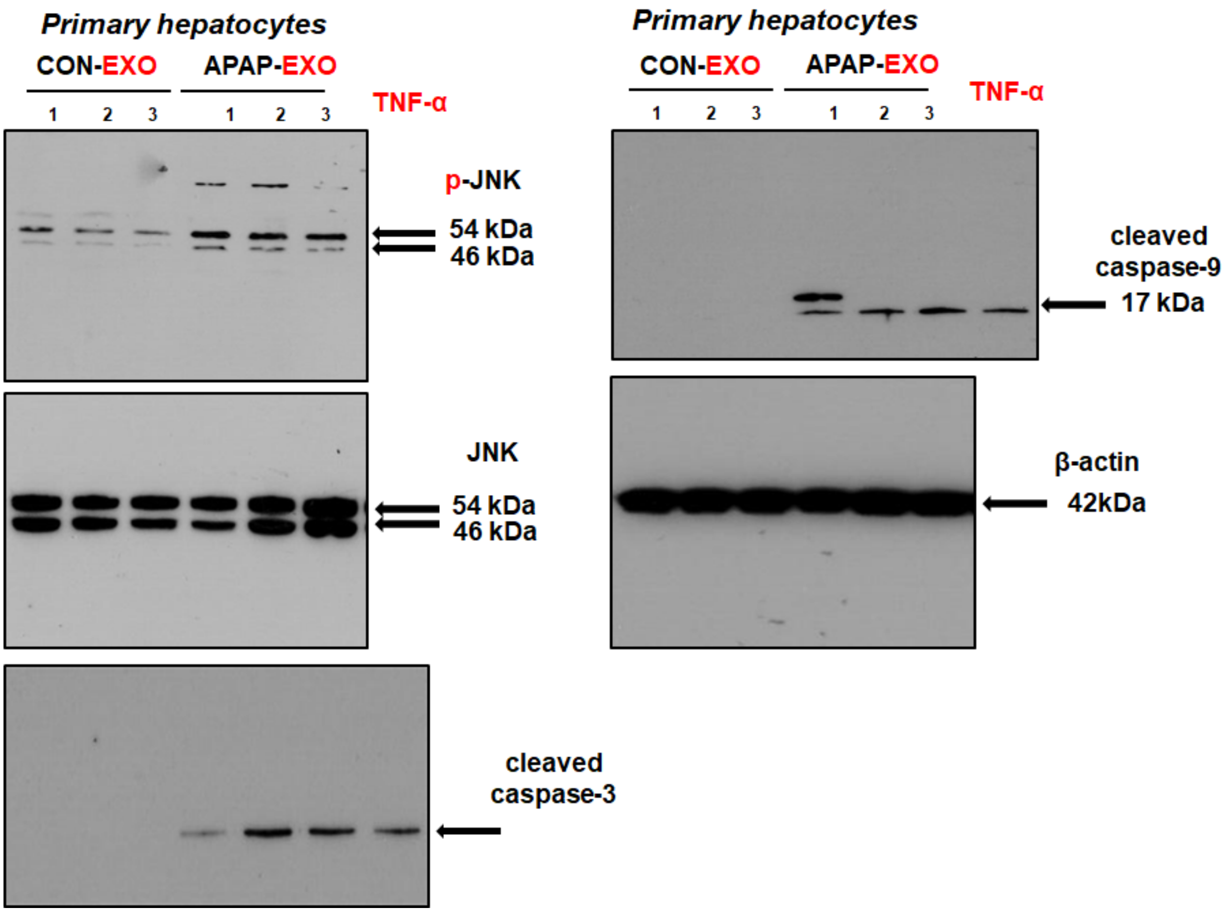

Supplementary Fig. 5. Full-length immunoblots for Fig. 5D.

Supplementary Fig. 6.

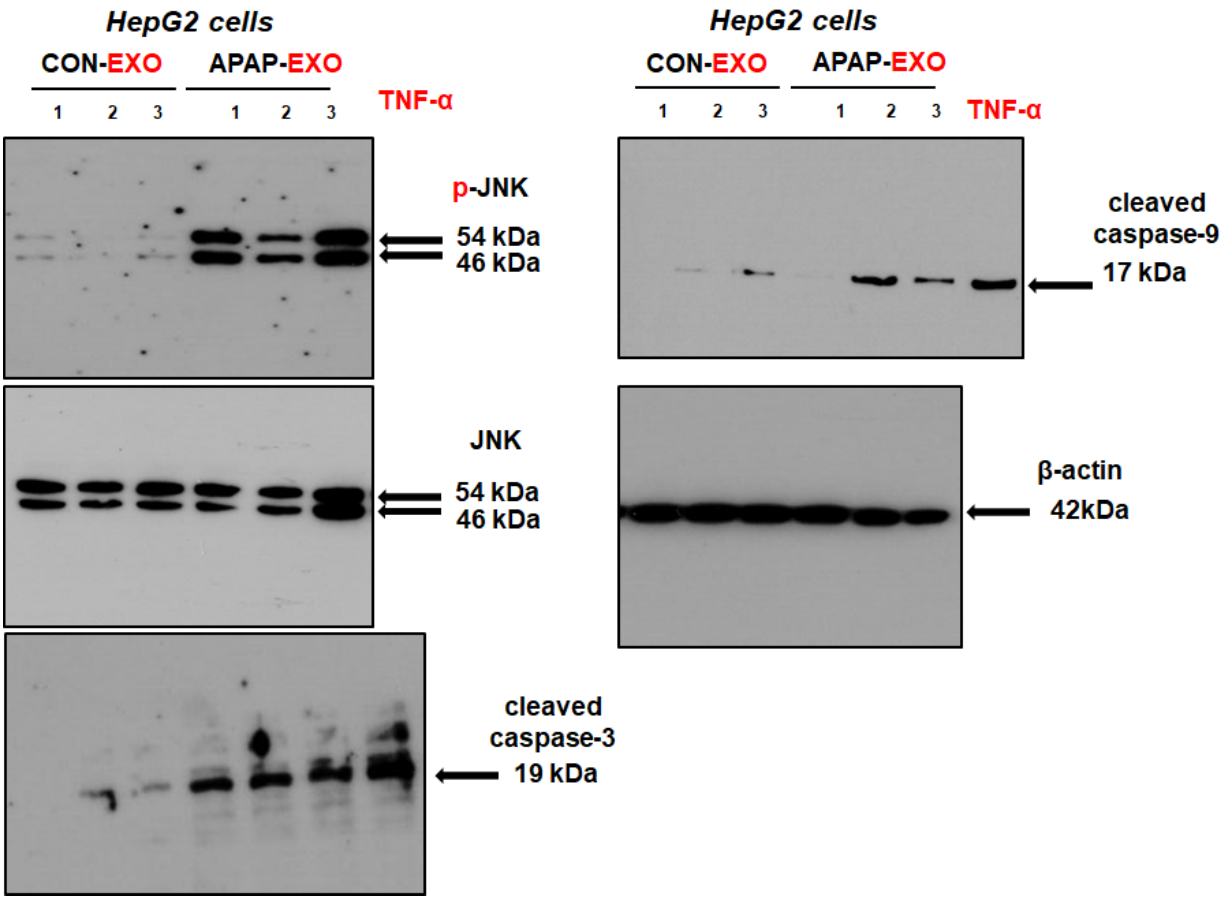

Supplementary Fig. 6. Full-length immunoblots for Fig. 5H.

Supplementary Fig. 7.

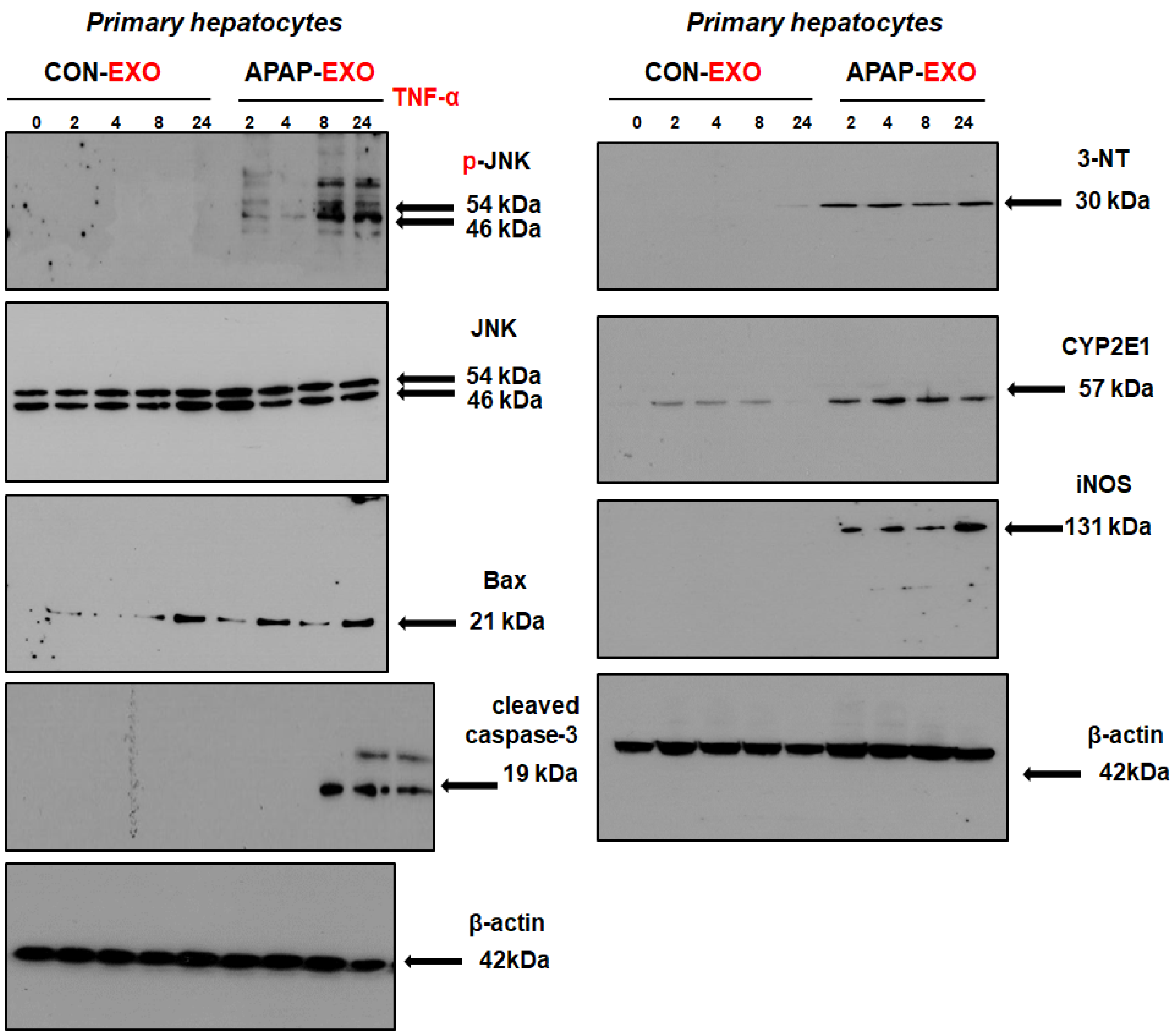

Supplementary Fig. 7. Full-length immunoblots for Fig. 6A and D.

Supplementary Fig. 8.

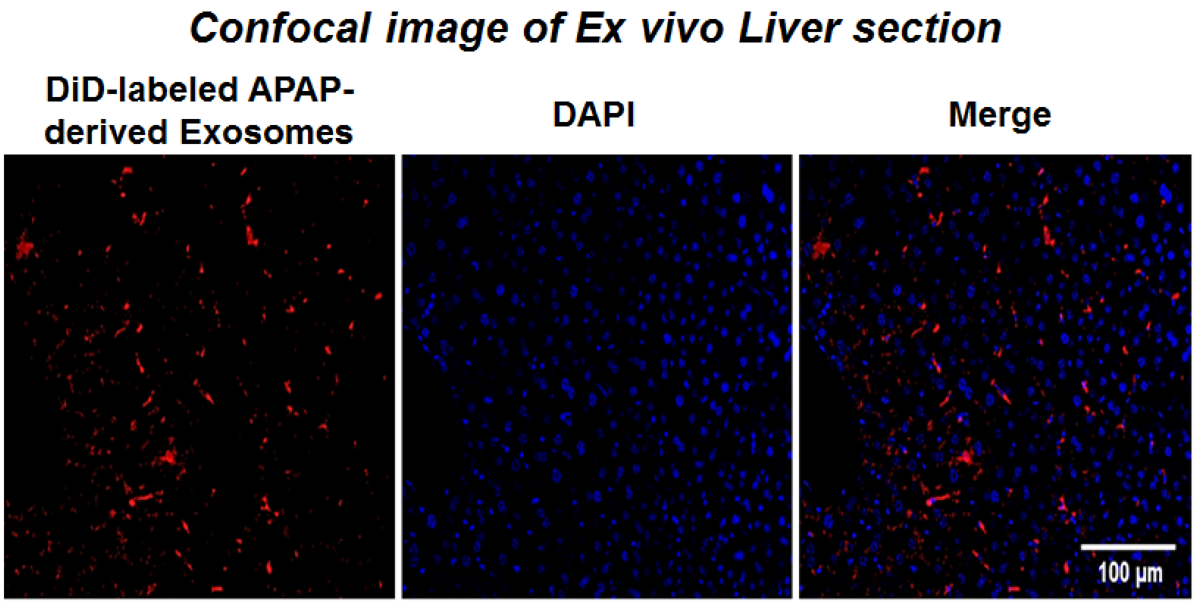

Supplementary Fig. 8. Cellular distribution of APAP-derived exosomes in mouse liver. Confocal microscopy image showing internalization of DiD-labeled exosomes into hepatocytes of *ex vivo* liver section. Cellular nuclei were counter-stained with DAPI. Scale bar = 100 µm.

Supplementary Fig. 9.

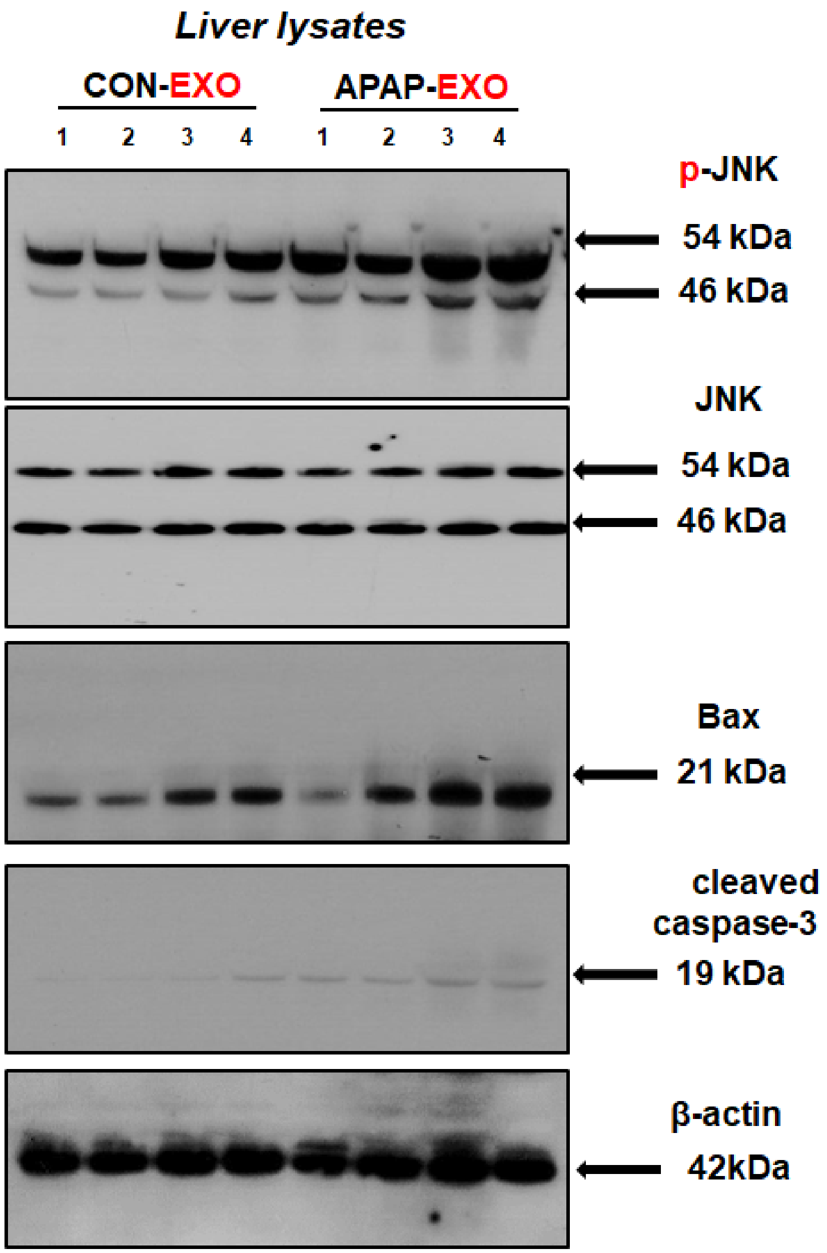

Supplementary Fig. 9. Full-length immunoblots for Fig. 7F.

Supplementary Fig. 10.

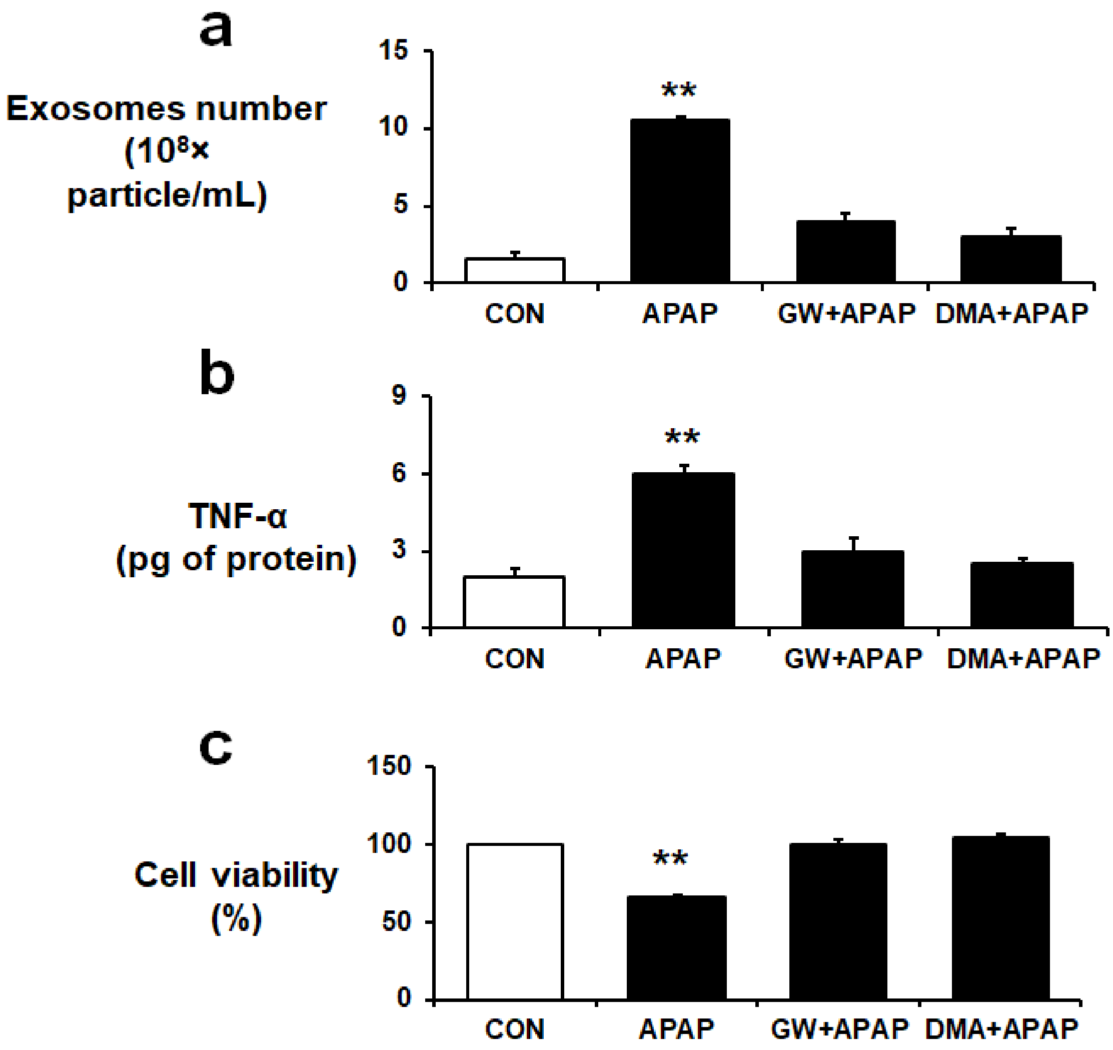

Supplementary Fig. 10. Exosomes number, TNF-α production and cell viability in APAP-induced hepatotoxicity after exposure to exosomes secretion inhibitors. Primary mouse hepatocytes were treated with 20 mM APAP in the absence or presence of 20 µM GW4869 or 25 µg/mL dimethyl amiloride (DMA) for 24 h. (A) Total number of exosomes in the each indicated group was measured by Nanosight. (B) the levels of TNF-α protein and (C) cell viability changes in the indicated groups (n=8/sample) are shown. **$P < 0.01$.
